# Geospatial mapping and risk factor analysis of *Leptospira interrogans* serovar Canicola Portland Vere in backyard cattle in Chiapas, Mexico

**DOI:** 10.14202/vetworld.2025.2636-2649

**Published:** 2025-09-11

**Authors:** Liliana del Rosario Velázquez Noriega, José Del Carmen Rejón-Orantes, Enrique Herrera López, José Luis Gutiérrez Hernández, Francisco Antonio Cigarroa Vázquez, Leopoldo Medina Sansón, Carlos Alfredo Carmona Gasca, José Ángel Gutiérrez Martínez, Rafael Enrique Ruiz Echeverría, Gerardo Uriel Bautista Trujillo

**Affiliations:** 1 Master of Science in Tropical Agropecuary Production, Meritorious Autonomous University of Chiapas, Tuxtla Gutiérrez, Chiapas, Mexico; 2 Chiapas Medicinal Plants Research Institute, Meritorious Autonomous University of Chiapas, Tuxtla Gutiérrez, Chiapas, Mexico; 3 National Center for Disciplinary Research in Animal Health and Safety- National Institute of Forestry, Agricultural, and Livestock Research, Palo Alto, Ciudad de Mexico; 4 Department of Biosafety and Food Safety, Mezcalapa School of Agricultural and Livestock Studies, Meritorious Autonomous University of Chiapas, Chiapas, Mexico; 5 Functional Biology Laboratory, Academic Unit for Veterinary Medicine and Zootechnics, Autonomous University of Nayarit, Tepic, Mexico; 6 Department of Animal Health and Safety, Faculty of Veterinary Medicine and Zootechnics CII, Meritorious Autonomous University of Chiapas, Tuxtla Gutierrez, Chiapas, Mexico

**Keywords:** backyard cattle, GIS mapping, *Leptospira*, Portland Vere, risk factors, seroprevalence, zoonosis, Chiapas, Mexico

## Abstract

**Background and Aim:**

Leptospirosis is a globally neglected zoonosis caused by pathogenic spirochetes of the genus *Leptospira*. It affects a wide range of animals and poses serious public and veterinary health risks. Backyard cattle systems, common across Latin America, are particularly vulnerable due to poor biosecurity and close animal–human–wildlife interfaces. This study assessed the seroprevalence, spatial distribution, and risk factors associated with *Leptospira* infection in backyard cattle herds of central Chiapas, Mexico.

**Materials and Methods:**

A cross-sectional study was conducted from January to September 2022 across five municipalities. A total of 590 serum samples were collected and analyzed using the microscopic agglutination test with six serovars. The geographical coordinates of production units (PUs) were recorded using a Global Positioning System (GPS), and spatial analysis was conducted with ArcGIS 10.5. Risk factor associations were evaluated through Chi-square tests and multivariate logistic regression using RStudio and Statistical Package for the Social Sciences software.

**Results:**

The overall seroprevalence of *Leptospira* was 27.72% (95% confidence interval [CI]: 23.97%–31.75%), with the Portland Vere serovar being predominant (22.89%). Cintalapa exhibited the highest municipal seroprevalence (61.75%; odds ratio [OR] = 6.2). Logistic regression identified significant risk factors for seropositivity, including artificial insemination (OR = 2.43), use of Jagüey reservoirs (OR = 0.47), and cattle aged 3 years (OR = 0.54) or 8 years (OR = 1.98). The American Swiss × Holstein crossbreed was significantly associated with increased seroprevalence (OR = 3.15). The presence of dogs within PUs was significantly associated with Portland Vere seropositivity (OR = 3.82), highlighting a possible role in disease transmission.

**Conclusion:**

This study highlights a high burden of *Leptospira interrogans* serovar Canicola Portland Vere in backyard cattle of central Chiapas. Key risk factors include specific breeding methods, water sources, age, and breed. The integration of serological surveillance, spatial mapping, and statistical modeling proved effective in identifying epidemiological hotspots and informing future One Health-based prevention strategies. The findings emphasize the need for enhanced disease surveillance, targeted control programs, and public health interventions tailored to small-scale cattle systems in tropical regions.

## INTRODUCTION

The backyard cattle system, also known as the small-scale livestock system, is a traditional model prevalent in many countries, including Mexico. This system typically involves livestock rearing on small plots of land, primarily for household subsistence. Despite its cultural and economic importance, limited technological integration and restricted access to veterinary services render these systems highly vulnerable to infectious diseases [1].

Among these, leptospirosis stands out as a particularly significant disease affecting cattle. Caused by pathogenic serovars of the genus *Leptospira*, the infection is frequently associated with reproductive disorders in cows, including abortion, infertility, and other clinical manifestations that impair productivity and elevate veterinary costs [2, 3]. Economic losses due to leptospirosis in cattle herds can range from 10% to as high as 85% of annual production, depending on several factors such as the infecting serovar, pathogen virulence, cattle breed, production system, management practices, preventive measures, and environmental conditions [4].

The World Health Organization classifies leptospirosis as a neglected tropical disease of major public health concern. Globally, approximately 10% of the population, particularly in tropical regions, is at risk of infection through direct or indirect contact with the urine of infected animals [5]. Clinically, leptospirosis may present in various forms: An acute septicemic phase, a severe icteric phase (also known as Weil’s disease), and a chronic phase. The icteric phase is the most severe and carries a mortality rate between 6% and 10% [5, 6].

At present, the genus *Leptospira* comprises 69 recognized species, over 300 serovars, and more than 25 serogroups [7]. In cattle, commonly reported serovars include Bratislava, Wolffi, Hardjo, Icterohaemorrhagiae, Canicola, and Tarassovi. The prevalence of *Leptospira*l infection in cattle varies significantly across geographic regions. For example, relatively low prevalence rates of 4.2% and 6.3% have been reported in Uganda [8] and Indonesia [9], respectively, whereas much higher rates, ranging from 60% to 81.8%, have been documented in Brazil’s Caatinga biome [10] and South Sudan’s Bahr El Ghazal region [11].

Tropical climates are strongly correlated with increased *Leptospira*l seroprevalence in cattle populations [12, 13]. A range of risk factors contribute to the persistence and transmission of *Leptospira*, especially in tropical and subtropical settings [14]. Environmental variables such as altitude, latitude [15], contaminated water sources [16], relative humidity, rainfall, and ambient temperature play critical roles [12]. In addition, herd-related factors, including size, management practices, grazing systems [17], and the presence of reservoir animals, particularly dogs, have been identified as important determinants of infection risk [18].

Despite the recognized public health significance and economic impact of leptospirosis in cattle, limited attention has been paid to its occurrence in smallholder or backyard production systems, particularly in Mesoamerican regions such as southern Mexico. Existing studies in Mexico and other tropical countries have primarily focused on large-scale or dual-purpose herds, overlooking the epidemiological complexity and zoonotic risk posed by backyard cattle, which often lack proper veterinary oversight and biosecurity. Moreover, while *Leptospira* serovars such as Hardjo, Icterohaemorrhagiae, and Pomona have been widely studied, there is a paucity of information on the spatial distribution and risk factors associated with *Leptospira* *interrogans* serovar Canicola Portland Vere in cattle. This knowledge gap is particularly concerning given the increasing evidence of cross-species transmission involving dogs, rodents, and livestock in shared environments. Furthermore, the role of local environmental variables (e.g., water source type, geographic clustering, age, and breed susceptibility) in the persistence and spread of *Leptospira* in tropical backyard systems remains insufficiently explored. To date, no published studies have systematically assessed the seroprevalence, spatial distribution, and multifactorial risk profile of *Leptospira*, specifically Portland Vere, in backyard cattle in Chiapas, a region characterized by high livestock density and ecological heterogeneity.

This study aimed to investigate the seroprevalence and spatial distribution of *L. interrogans* in backyard cattle systems in central Chiapas, Mexico, with a particular focus on the Portland Vere serovar. It also sought to identify and analyze the epidemiological, environmental, and management-related risk factors associated with *Leptospira* infection. Through the integration of serological diagnostics (microscopic agglutination test[MAT]), geospatial mapping (ArcGIS), and multivariate statistical modeling (Statistical Package for the Social Sciences [SPSS] and R), the study provides a comprehensive evaluation of disease burden and transmission dynamics within an underrepresented livestock system. The findings are intended to inform targeted surveillance, biosecurity enhancement, and One Health-based control strategies for leptospirosis in tropical and subtropical settings.

## MATERIALS AND METHODS

### Ethical approval

The Ethics Committee of the University of Sciences and Arts of Chiapas approved the study protocol (Approval No. 049/02-2018).

### Study period and location

Between January and September 2022, 590 blood serum samples were collected from cattle in 38 backyard production units (PUs) across five municipalities in central Chiapas, Mexico (Cintalapa, Jiquipilas, Ocozocoautla, San Fernando, and Suchiapa). The study region features a warm, humid climate with summer rainfall, an average annual temperature of 30°C, and precipitation ranging from 787 to 1,703 mm. The region’s natural vegetation primarily consists of lowland deciduous forests. The region encompasses several protected natural areas, including the El Ocote Biosphere Reserve, the La Perla Ecological Conservation Area, and the Sumidero Canyon National Park. It also includes part of the Grijalva River basin, which houses the hydroelectric plants of the Nezahualcoyotl and Manuel Moreno Torres.

### Selection of PUs and management characteristics

PUs and cattle were selected based on owner consent for data use and sampling, convenience sampling, and ethical handling, with a focus on backyard livestock systems. These backyard systems featured mixed feeding practices, with limited pasture areas comprising grasses (*Megathyrsus maximus*, *Hyparrhenia rufa*, and *Cynodon nlemfuensis*) and legumes (*Mimosa* spp. and *Leucaena leucocephala*). Feed supplementation included ground corn or sorghum and mineral salts (bovine salt) as available. Wooden posts were generally used to construct corrals and were shaded by trees or metal roofing sheets.

### Study design and risk factor data collection

Data were collected through a structured questionnaire, including locality, breed, age, feeding type, reproductive status, reproductive disorders, water source, breeding method, and vector presence. Reproductive factors (non-pregnant cows, pregnant cows, artificial insemination, natural mating, abortion, fetal mummification, stillbirth, retained placenta, and repeated estrus), environmental factors (Jagüey, wells, and stream), cattle age (3–13 years), cattle breed (American Swiss/Holstein, American Swiss, Brangus, Gyrolando, Charolais, Zebu/Simmental, Zebu/Brahman, Simmental, Simbrah, Zebu/American Swiss, Zebu/Simbrah, Zebu/Charolais, Brahman, Zebu/Holstein, Sardo Negro, and Zebu/Zebu), vectors (presence of dogs, presence of rodents, and shares a bull), and feeding systems (grazing and semi-stabled) were evaluated as risk factors for *Leptospira*.

PUs were included if they contained breeding-age female cattle aged 3 years. Cows displaying clinical signs such as abortion, retained placenta, fetal mummification, stillbirth, repeated estrus, infertility, or anestrus were recorded where applicable. The breeding bulls present in the herds were also sampled. The presence of dogs and cats, as well as rats or mice, was documented at each site. Herds with a history of vaccination against *Leptospira* or containing sexually immature cattle were excluded from the study.

### Sample collection and laboratory diagnosis

Five milliliters of blood was drawn from each animal and left standing upright in the shade at room temperature (30°C) for 20 min to allow serum separation. The coccygeal vein, located between the second and seventh coccygeal vertebrae along the ventral midline, was used to obtain samples. Vacutainer tubes (6 mL, BD, USA) without anticoagulant and pre-sealed under vacuum were used for blood collection. Each collection tube was pre-labeled with the corresponding animal’s ear tag number. Blood was collected using a 21-gauge, 1.5-inch needle and a sterile plastic tube holder. The samples were transported to the Leptospirosis Laboratory at the National Research Center for Animal Health and Safety (CENID-SAI), Mexico City, under the National Institute for Forestry, Agricultural and Livestock Research (INIFAP). Serum was separated by centrifuging the samples at 3,000 × *g* for 10 min and then stored in polystyrene microtubes at −4°C until analysis.

*Leptospira* was diagnosed using the reference standard MAT with live antigens, employing a panel of six serovars most prevalent in Mexico [19]. The panel comprised three international reference strains (Bratislava, Wolffi, and Tarassovi) and three Mexican isolates (Hardjoprajitno, Icterohaemorrhagiae, and Portland Vere) (Table 1).

**Table 1:** Strains used for diagnosis with Microscopic Agglutination Test (MAT).

Species	Serogroup	Serovar	Strains
*L. interrogans*	Australis	Bratislava	Jes-Bratislava
*L. interrogans*	Sejroe	Wolffi	3707
*L. borgpetersenii*	Tarassovi	Tarassovi	Perepelitsin
*L. interrogans*	Sejroe	Hardjoprajitno	H-89*
*L. interrogans*	Icterohaemorrhagiae	Icterohaemorrhagiae	Palo Alto*
*L. interrogans*	Canicola	Portland Vere	Sinaloa ACR*

* Strains isolated in Mexico. *L. interrogans = Leptospira interrogans, L. borgpetersenii = Leptospira borgpetersenii*

Serum samples were initially diluted 1:25 and subsequently serially diluted twofold to a final dilution of 1:1600. Agglutination results were evaluated using a dark-field microscope at 100× magnification. The antibody titer was defined as the highest serum dilution showing at least 50% agglutination of *Leptospira* organisms. Samples were classified as positive if they exhibited ≥50% agglutination. Samples with a titer of 1:50 were considered suspicious, whereas those with titers ranging from 1:100 to 1:600 were deemed seropositive, indicating a positive test result for *Leptospira* antibodies. Samples exhibiting no agglutination were classified as negative.

### Geospatial data acquisition and mapping

The NoteCam Lite GPS Camera application (version 5.15, Derekr Corp., India) was used to record the geographical coordinates of municipal centers and individual PUs, with an accuracy of approximately ±5 meters. ArcGIS software (version 10.5, CA, USA) was employed to generate maps depicting the spatial distribution of *Leptospira* seroprevalence across the five municipalities under study. Mapping was conducted using the exact coordinates of each PU, combined with the prevalence data for the six identified *Leptospira* serovars at each location [20].

### Data management and statistical analysis

Survey responses and laboratory results were entered into an electronic database using Microsoft Excel. Descriptive statistics and prevalence analyses were conducted using RStudio [21] with R version 4.2.2. The spatial visualization of the results was performed using ArcGIS software version 10.5 [20]. Risk factor identification and logistic regression analysis were performed using IBM SPSS Statistics software version 25 [22].

All serum samples that tested positive for at least one serovar were included in the analysis to estimate the prevalence of *Leptospira* serovars. The true prevalence, defined as the true proportion of infected individuals in a population, was calculated using the Rogan–Gladen estimator [23]. Confidence intervals (CIs) were computed using the method proposed by Reiczigel *et al*. [24]. Serovar-specific prevalence analysis was performed using the “epiR” package in R [25]. Heatmaps for spatial visualization were generated using the “superheat” package [26] within R software [21].

Statistical associations between *Leptospira* seropositivity (dependent variable) and independent variables, including locality, breed, age, feeding type, reproductive status, reproductive disorders, water source, breeding method, and vector presence, were assessed. Chi-square tests (χ^2^) were applied to 2 × 2 contingency tables to assess significance, using a p-value threshold of <0.05. Odds ratios (ORs) with corresponding 95% CIs were calculated. The variables that were statistically significant in the χ^2^ analysis were included in a multivariate logistic regression to explore potential interactions. Variables with a p-value of 0.2 were entered into a stepwise logistic regression model. The goodness-of-fit of the model was evaluated using the Hosmer–Lemeshow test (p > 0.05), and statistically significant predictors (p < 0.05) were retained [27]. Bivariate and multivariate analyses were conducted using IBM SPSS Statistics version 25 [22].

## RESULTS

### Overall seroprevalence of *Leptospira* spp.

A total of 590 blood serum samples were collected from 38 PUs across five municipalities. The MAT results showed an overall *Leptospira* seroprevalence of 27.72% (95% CI = 23.97%–31.75%), with 89.47% (34/38) of the PUs testing positive. The most prevalent *Leptospira* serovar was Canicola Portland Vere, detected in 22.89% of samples (95% CI = 19.36%–26.75%; p ≤ 0.05; OR = 3.2), followed by Bratislava at 5.51% (95% CI = 3.36%–8.21%) (Table 2).

**Table 2:** Prevalence of *Leptospira* serovars in backyard cattle (N = 590) from the central zone of Chiapas, Mexico.

Serovars	P-value	N	Frequency (%)	Prevalence (%)	CI (95%)
Portland Vere	149	441	25.25	22.89*	19.36–26.75
Bratislava	52	538	8.81	5.51	3.36–8.21
Icterohaemorrhagiae	11	579	1.86	1.83	2.70–0.30
Hardjoprajitno (H-89)	7	583	1.18	2.55	3.19–1.23
Tarassovi	3	587	0.50	3.27	3.62–2.23
Wolffi	1	589	0.16	1.13	0.24–1.54

* Statistically significant p < 0.05: test χ²; p *=* Positive; N = Negative, CI = Confidence intervals

### Spatial distribution of *Leptospira* serovars in the study region

**Figure 1:**
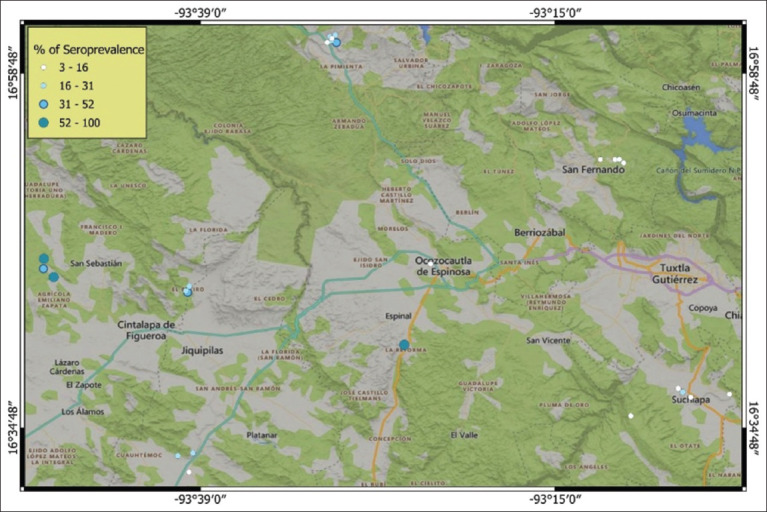
Heat map of five municipalities of the central zone of Chiapas showing the seroprevalence of *Leptospira* spp. [Source: Original elaboration by the authors, base map available under the Open Database License ©OpenStreetMap].

MAT results indicated that 89.47% (34 of 38) of the analyzed PUs were seropositive, defined as units with at least one animal testing positive for *Leptospira* antibodies. The municipality of Cintalapa exhibited the highest seroprevalence at 61.75% (80/129; 95% CI = 52.65%–70.11%), which was significantly associated with exposure to *Leptospira* (p ≤ 0.05; OR = 6.2). Ocozocoautla reported a seroprevalence of 30.29% (40/124; 95% CI = 22.27%–39.44%) without statistical significance (p ≥ 0.05; OR = 1.1). Other municipalities showed lower prevalence rates: Jiquipilas (19.97%; 95% CI = 13.05%–28.70%; p ≤ 0.05; OR = 0.62), San Fernando (11.44%; 95% CI = 5.64%–19.91%; p ≤ 0.05; OR = 0.34), and Suchiapa (9.29%; 95% CI = 4.14%–17.04%; p ≤ 0.05; OR = 0.27) (Figure 1).

**Figure 2:**
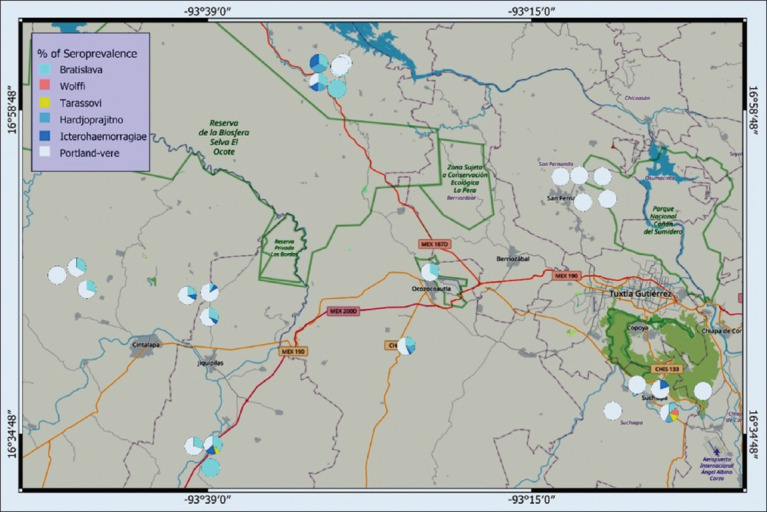
Spatial distribution map of the identified *Leptospira* serovars in five municipalities of the central zone of Chiapas, Mexico [Source: Original elaboration by the authors, base map from Bing Maps, available under the Open Database License ©Microsoft Corporation].

Portland Vere was detected in all municipalities, showing the highest prevalence in Cintalapa (30%–100%), followed by Ocozocoautla (8%–80%). Bratislava seroprevalence peaked in Cintalapa (10%–42.3%), followed by Ocozocoautla (6.6%–33.3%) and Jiquipilas (3.44%–20%). Icterohaemorrhagiae had the highest prevalence in Ocozocoautla (5.5%–10%), followed by Jiquipilas (5%–5.8%) and Cintalapa (2.9%–5%). Hardjoprajitno was most prevalent in Ocozocoautla (10%–22.2%) and also identified in Suchiapa (5.26%). Tarassovi and Wolffi were only detected in Suchiapa (5.26%), with Tarassovi also appearing in Jiquipilas (2.94%) (Figure 2).

### Association between seroprevalence and management, reproductive, and environmental factors

Survey data showed that 40.9% (9/22) of cows with a history of reproductive disorders, including repeated estrus, retained placenta, anestrus, abortion, stillbirth, and fetal mummification, were seropositive for at least one *Leptospira* serovar. In contrast, 29.8% of the 543 cows without reproductive issues tested positive.

Significant associations (p < 0.05) were found between *Leptospira* seroprevalence and the use of artificial insemination (OR = 2.1; CI = 1.19%–3.87%), Jagüey water reservoirs (OR = 0.4; CI = 0.23%–2.47%), and wells (OR = 1.72; CI = 1.20%–2.47%).

No significant association was detected between seroprevalence and reproductive status – non-pregnant cows (OR = 1.36; CI = 0.95%–1.93%) or pregnant cows (OR = 0.79; CI = 0.55%–1.13%). However, cows with a history of abortion showed an increased risk of infection (OR = 2.39; CI = 0.68%–8.36%) (Table 3).

**Table 3:** Association of *Leptospira* seropositivity with management, reproductive, and environmental factors in cattle from the central zone of Chiapas, Mexico.

Factors	Samples	Positive	Frequency (%)	p-value	OR	CI (95%)
Non-pregnant cows	280	93	33.2	0.8	1.36	0.95–1.93
Pregnant cows	285	78	27.3	0.2	0.79	0.55–1.13
Bulls	25	5	20	0.2	0.57	0.21–1.56
Artificial insemination	50	23	46	0.009*	2.1	1.19–3.87
Natural mating	459	132	28.8	0.28	0.79	0.52–1.20
Artificial insemination and natural mating use	81	21	25.9	0.40	0.79	0.67–1.36
Abortion	10	5	50	0.16	2.39	0.68–8.36
Fetal mummification	1	1	100	0.29	1	0.99–1.76
Stillbirth	1	1	100	0.29	1	0.99–1.01
Retained placenta	2	0	0	0.35	0.99	0.98–1.00
Repeated estrus	7	2	28.6	0.94	0.94	0.18–4.89
Jagüey	109	18	16.51	0.00*	0.40	0.23–2.47
Wells	215	80	37.20	0.03*	1.72	1.20–2.47
Stream	266	78	29.32	0.80	0.95	0.67–1.36

* Statistically significant p < 0.05: test χ², F = Frequency, OR = Odds ratio, CI = Confidence intervals

Portland Vere seropositivity was significantly associated with artificial insemination (p < 0.05; OR = 2.15; CI = 1.32%–3.58%), while Hardjoprajitno was associated with natural mating (p < 0.05; OR = 0.20; CI = 0.22%–0.95%). Abortion in cows was linked to increased risk, particularly with the serovars Hardjoprajitno (p > 0.05; OR = 10.6; CI = 8.21%–16.7%), Icterohaemorrhagiae (p > 0.05; OR = 6.33; CI = 4.87%–8.66%), Bratislava (p > 0.05; OR = 2.6; CI = 1.34%–4.91%), and Portland Vere (p > 0.05; OR = 2.0; CI = 1.11%–3.66%), though these associations were not statistically significant.

Repeated estrus was associated with Hardjoprajitno (OR = 16.02; CI = 11.21%–26.8%) and Icterohaemorrhagiae (OR = 9.55; CI = 7.21%–15.6%), although neither was statistically significant (p > 0.05). Both Portland Vere and Bratislava showed significant associations (p < 0.05) with Jagüey water use (OR = 0.48 and 0.79) and well water (OR = 1.61 and 1.84), though these findings were not tabulated.

### Association between seroprevalence and cattle age

A significant association was observed between *Leptospira* seroprevalence and cattle aged 3 years (OR = 0.50; 95% CI = 0.28–0.90) and 8 years (OR = 2.15; 95% CI = 1.13–4.09). In addition, 12-year-old cows exhibited a higher odds ratio (OR = 4.79; 95% CI = 0.86–24.40), although this was not statistically significant (Table 4).

**Table 4:** Relationship between *Leptospira* seropositivity and cattle age (N = 590) in central Chiapas, Mexico.

Age (years)	Samples	Positive	Frequency (%)	p-value	OR	CI (95%)
3	86	16	18.60	0.01*	0.50	0.28–0.90
4	145	35	24.13	0.08	0.68	0.44–1.05
5	128	43	33.59	0.23	1.28	0.84–1.96
6	97	29	29.89	0.95	0.98	0.61–1.58
7	49	20	40.81	0.06	1.76	0.96–3.23
8	41	19	46.34	0.01*	2.15	1.13–4.09
9	13	5	38.46	0.49	1.48	0.47–4.60
10	21	5	23.80	0.53	0.72	0.26–2.01
11	3	0	0.00	0.28	0.99	0.98–1.00
12	6	4	66.66	0.06	4.79	0.86–24.40
13	1	0	0.00	0.51	0.99	0.99–1.00

* Statistically significant p < 0.05: test χ², OR = Odds ratio, CI = Confidence intervals

Portland Vere was significantly associated (p < 0.05) with seropositivity in cattle aged 12 (66.67%), 8 (46.34%), and 3 years (15.11%). Grouped age analysis also revealed significant associations between *Leptospira* infection, especially Portland Vere, and cattle aged 3–4 and 7–8 years.

### Seroprevalence in purebred versus crossbred cattle

The study evaluated eight crossbreeds (e.g., Zebu/Brahman, Zebu/Zebu, Zebu/Charolais, Zebu/Holstein, Zebu/Simbrah, Zebu/Simmental, Zebu/American Swiss, and American Swiss/Holstein) and eight pure breeds (e.g., Brahman, Brangus, Charolais, Gyrolando, Sardo Negro, Simbrah, Simmental, and American Swiss). Overall, *Leptospira* seroprevalence was higher in purebred cattle (30.05%) than in crossbreeds (27.08%).

A statistically significant association was observed with the American Swiss/Holstein crossbreed, which exhibited an odds ratio of 11.1 (Table 5). Portland Vere was strongly associated with this breed (p < 0.05; OR = 5.39; CI = 4.25%–7.66%), with a seroprevalence of 66.30%.

**Table 5:** Relationship between *Leptospira* seropositivity and cattle breed (N = 590) in central Chiapas, Mexico

Breed	Sample	Positive	Frequency (%)	p	OR	CI (95%)
American Swiss/Holstein	11	9	81.81	0.00*	11.10	2.37–51.97
American Swiss	38	15	39.47	0.06	1.93	0.96–3.90
Brangus	1	1	100	0.12	1.0	0.99–1.01
Gyrolando	1	1	100	0.12	1.0	0.99–1.01
Charolais	4	0	0	0.19	0.99	0.98–1.00
Zebu/Simmental	59	14	23.72	0.24	0.69	0.37–1.29
Zebu/Brahman	3	0	0	0.25	0.99	0.98–1.00
Simmental	3	0	0	0.25	0.99	0.98–1.00
Simbrah	4	2	50	0.37	2.36	0.33–16.94
Zebu/American Swiss	378	109	28.83	0.51	0.88	0.61–1.27
Zebu/Simbrah	1	0	0	0.51	0.99	0.98–1.00
Zebu/Charolais	2	1	50	0.53	2.36	0.14–37.94
Brahman	5	1	20	0.62	0.58	0.06–5.27
Zebu/Holstein	8	2	25	0.76	0.78	0.15–3.91
Sardo Negro	4	1	25	0.83	0.78	0.08–7.57
Zebu/Zebu	68	20	29.41	0.93	0.97	0.59–1.70

* Statistically significant p < 0.05: test χ², OR = Odds ratio, CI = Confidence intervals

Seroprevalence was also significantly associated with the presence of dogs (OR = 7.5; CI = 0.81%–69.07%) and grazing practices (OR = 8.50; CI = 3.38%–21.34%) (Table 6). Portland Vere serovar showed a strong association with dog presence (p < 0.05; OR = 3.82; CI = 1.20%–7.64%) and a negative association with rodents (OR = 0.57; CI = 0.11%–2.34%). Similarly, Bratislava (OR = 0.41; CI = 0.11%–2.74%) and Hardjoprajitno (OR = 0.16; CI = 0.11%–1.34%) showed links to rodent presence, although these were not included in the table.

**Table 6:** Association of *Leptospira* seropositivity with vectors and feeding systems per production unit (N=38) in cattle from the central zone of Chiapas, Mexico.

Factors	Positive	Frequency (%)	p	OR	CI (95%)
Presence of dogs	30	78.94	0.04*	7.50	0.81–69.07
Grazing	30	78.94	0.04*	8.50	3.38–21.34
Presence of rodents	26	68.42	0.25	3.25	0.39–26.91
Shares a bull	4	10.52	0.46	1.13	1.00–1.28
Semi-stabled	4	10.52	0.45	0.40	0.03–4.83

* Statistically significant p < 0.05: test χ², F = Frequency, OR = Odds ratio, CI = Confidence intervals

### Risk factors identified through logistic regression analysis

Following bivariate screening, logistic regression analysis was performed, with model adequacy confirmed using the Hosmer–Lemeshow goodness-of-fit test (p > 0.05). Key risk factors associated with *Leptospira* seropositivity included the American Swiss × Holstein crossbreed, use of Jagüey water reservoirs, artificial insemination, and cattle aged 3 or 8 years (Table 7).

**Table 7:** Risk factors associated with *Leptospira* seropositivity in cattle from the central zone of Chiapas, Mexico.

Factors	B	p	OR	CI (95%)
American Swiss/Holstein	1.322	0.01*	3.15	1.30–10.76
Jagüey	−0.741	0.01*	0.47	0.27–0.84
Artificial insemination	0.889	0.01*	2.43	1.15–5.13
3-year-old bovine	−0.615	0.03*	0.54	0.30–0.96
8-year-old bovine	0.685	0.03*	1.98	1.04–3.78
American Swiss	0.680	0.06	1.97	0.97–4.01
Wells	0.356	0.06	1.42	0.97–2.09

* Statistically significant p < 0.05: Logistic regression analysis, B = Regression coefficient, OR =Odds ratio, CI = Confidence intervals

For the Portland Vere serovar, significant associations were found with residence in Cintalapa (B = 1.607; OR = 4.99; CI = 2.87%–8.66%), reduced risk in Suchiapa (B = −0.671; OR = 0.467; CI = 0.22%–0.95%), and increased odds in 8-year-old cattle (B = 0.943; OR = 2.57; CI = 1.34%–4.91%). For Bratislava, risk factors included lower odds in Suchiapa (B = −2.592; OR = 0.075; CI = 0.11%–0.15%) and higher odds in Cintalapa (B = 0.701; OR = 2.02; CI = 1.11%–3.66%), although these results were not tabulated.

## DISCUSSION

### Epidemiological significance of backyard cattle systems in Latin America

Backyard or small-scale cattle herding systems are prevalent in Mexico and across Central and South America. These systems significantly contribute to local economic resilience and the preservation of cultural practices. However, they often suffer from low productivity due to inadequate infrastructure and limited access to veterinary services, which restrict effective disease prevention and control measures.

### Study novelty and regional relevance

This is the first study to establish associations between *Leptospira* seroprevalence and specific risk factors in sub-humid tropical backyard cattle systems. The study area holds particular importance due to its intensive cattle trade, encompassing diverse purebred and crossbred livestock representative of broader Latin American production models. In addition, the region includes ecologically sensitive areas, such as protected natural zones and hydrological basins that serve as key ecological corridors.

### Seroprevalence and dominant *Leptospira* serovars

Overall, *Leptospira* seroprevalence was found to be 27.72%, with 89.47% of PUs registering at least one seropositive animal. The dominant serovar identified was *L. interrogans* Canicola Portland Vere (22.89%), followed by *L. interrogans* Australis Bratislava (5.51%). Spatial analysis revealed considerable geographic variability in the distribution of different *Leptospira* serovars.

### Global and national context of *Leptospira* prevalence

The global seroprevalence of *Leptospira* in cattle shows broad variability. In Africa, reported rates range from 4.2% in Uganda [8] to 81.8% in South Sudan [11]. In Asia, prevalence ranges from 6.32% in Indonesia [9] to 41.4% in India [28], with diverse serovars, such as Icterohaemorrhagiae, Pomona, and Javanica being common. In the Americas, the prevalence varies widely. Colombia reports 34.2%–39.9% [29], Ecuador up to 53.3% [30], and Brazil from 39.8% to 83.3% [31], depending on herd size and reproductive management. Mexican reports show a range from 8.57% to 48.1%, with regional differences influenced by production systems and diagnostic methods.

### Comparative data from other Mexican states

Studies in Oaxaca identified Hardjoprajitno (49.09%) and Icterohaemorrhagiae (34.89%) as dominant serovars [19]. Veracruz reported even higher frequencies for Icterohaemorrhagiae (71.3%) and Hardjoprajitno (91.8%), while Puebla and Tabasco identified Wolffi and the Palo Alto strain of Icterohaemorrhagiae, respectively, as predominant [32]. In Chiapas, Sánchez *et al*. [33] documented a 63% seroprevalence in the municipalities of Tecpatán and Juárez, with Tarassovi and Icterohaemorrhagiae being most common. Another study in Veracruz municipal slaughterhouses reported 67.5% seroprevalence, with Tarassovi, Mini, and Hardjoprajitno as leading strains [34]. These inter-regional differences likely reflect environmental, management, and diagnostic variability.

### Geospatial clustering and environmental risk factors

This study confirms elevated *Leptospira* seroprevalence in Cintalapa and Ocozocoautla, Chiapas, regions with humid subtropical climates, abundant water sources, and dense livestock populations. Similar geographic clustering has been observed in other humid tropical areas, where natural water sources likely aid in the bacterium’s persistence and transmission.

### Key risk factors contributing to *Leptospira* seropositivity

Our risk factor analysis identified artificial insemination, dog presence, and the use of Jagüey or well water as significant predictors of increased *Leptospira* seropositivity. These findings align with previous studies by Andrade *et al*. [35] and Chong *et al*. [36] that have emphasized the role of water sources and domestic animals in facilitating *Leptospira* transmission.

### Significance of Canicola serovar and role of dogs

Although reports on the Canicola serovar in cattle are limited, Valdiviezo *et al*. [30] found Canicola to be the most prevalent serovar in southern Ecuador, detected in 15.17% of 389 cattle. Other studies confirm the serovar’s prevalence in dogs in Mexico [18, 37] and Brazil [31]. Transmission often occurs through contact with infected urine, particularly in grazing areas where interspecies sniffing behavior facilitates spread [35].

This mechanism may explain the notable presence of *L. interrogans* serogroup Canicola Portland Vere in cattle sampled in our study. Notably, 79% of the PUs in our study area had dogs present, which are recognized as maintenance hosts for this serovar [38, 39]. However, additional studies are necessary to evaluate *Leptospira* seroprevalence in regional dog populations, implement longitudinal surveillance, and investigate dog-to-cattle transmission dynamics.

### Contribution of other carrier species to environmental spread

*L. interrogans* has been identified in other animal reservoirs, including the vampire bat *Desmodus rotundus* [36], goats [40], captive wildlife [41], and rodents [42, 43]. These species may play an important role in maintaining and spreading *Leptospira* in shared environments, contributing to the risk of environmental contamination and cross-species infection.

### Geographic influence on serovar distribution

The highest frequencies of Portland Vere and Bratislava serovars were observed in Cintalapa and Ocozocoautla. Although no prior studies have been conducted in central Chiapas, these municipalities report the highest cattle populations (ranging from 67,641 to 153,934 head) and milk production (5,486.3 to 20,155.85 liters). Spatial variation in serovar prevalence may be influenced by factors such as urbanization, cattle movement to collection centers, inadequate breeding practices, and the presence of maintenance hosts [5, 37].

### Ecological risk to wildlife, domestic animals, and humans

The spatial distribution map revealed that *Leptospira*-positive herds were often located near protected natural areas and hydrological basins, posing potential risks to wildlife, domestic animals, and human populations [44]. The seroprevalence of *Leptospira* spp. in Chiapas illustrates the relevance of a One Health approach. Comprehensive integration of epidemiological, clinical, and environmental data is necessary to address its impacts on public health, food security, and ecological conservation [5].

### Reproductive disorders and serovar associations

This study revealed a higher frequency of *Leptospira* infection and increased odds ratios among cows with a history of abortion and other reproductive abnormalities. Reproductive issues such as abortion and repeated estrus were linked to heightened risk of *Leptospira* infection, notably with the Hardjoprajitno and Icterohaemorrhagiae serovars. These findings reinforce the reproductive pathogenicity well documented for these serovars.

### Other potential infectious contributors to reproductive disorders

It is important to note that reproductive complications may also arise from other pathogens, including Brucella *spp*., bovine viral diarrhea virus, infectious bovine rhinotracheitis, *Babesia* *spp*., *Neospora* *spp*., *Anaplasma* *spp*., and *Listeria* *spp*. [45]. Among the PUs analyzed, vaccination against these diseases was not reported, except for brucellosis, which is regulated under Mexico’s national control and eradication campaign [46].

### Age-related susceptibility to *Leptospira* infection

A statistical association was observed between *Leptospira* infection and cattle aged 3 and 8 years, consistent with prior studies by Guzmán *et al*. [47] and Yupiana *et al*. [48]. This could be due to increased movement, stress from crowding, and lack of vaccination, which heightens infection risk in younger animals. Older breeding cows may have had prior exposure and chronic infections, which sustain transmission within herds [49].

### Influence of breed and genetic resistance

This study also identified elevated seroprevalence in cows aged 3, 8, and 12 years, particularly for the Portland Vere serovar. These trends may indicate cumulative environmental exposure or age-related immune variations. Similar findings have been reported [45, 47], though the mechanisms remain unclear. This is the first study to report the American Swiss × Holstein crossbreed as a risk factor for *Leptospira* in herds from humid tropical zones. Prevalence by breed showed higher seropositivity in pure breeds (30.05%) than in crossbreeds (27.08%). This may be attributed to the hardiness conferred by *Bos taurus* × *Bos indicus* crosses, which enhances resistance to disease and harsh climates [9].

### Breed-specific associations with Portland Vere

The American Swiss × Holstein crossbreed, particularly in relation to the Portland Vere serovar, showed a strong association with *Leptospira* seropositivity. This may result from breed-specific susceptibility or distinct management practices. Prior studies by Alinaitwe *et al*. [8] and Onafruo *et al*. [11] suggest that some cattle breeds may exhibit heightened vulnerability due to genetic, immunological, or physiological factors. Further research is warranted to investigate the potential resistance or susceptibility of different breeds to *Leptospira*.

### Environmental factors driving *Leptospira* distribution

Spatial analysis is a valuable tool for identifying high-risk areas and environmental conditions favoring disease transmission. *Leptospira* was found to be widely distributed in municipalities with higher average annual rainfall, particularly Cintalapa (1,206.7 mm) and Ocozocoautla (2,000.3 mm). Humidity plays a critical role in the environmental survival of *Leptospira*, as the bacterium can persist for several weeks in stagnant water sources [16]. In addition, the study area maintains optimal temperature ranges for *Leptospira* growth (25°C–30°C), which further facilitates bacterial persistence and dissemination [50]. This finding supports the correlation between leptospirosis seroprevalence and environmental parameters such as rainfall and ambient temperature [12].

### Host and management-related factors from bivariate analysis

Bivariate analysis demonstrated a statistically significant association between *Leptospira* infection and several variables: animal age, breed, breeding method, water source, and the presence of dogs. These conditions support the continued occurrence of leptospirosis and are particularly linked to the Portland Vere and Bratislava serovars. The environmental characteristics of central Chiapas, characterized by a sub-humid tropical climate, water-rich terrain, and dense livestock activity, create ideal conditions for *Leptospira* survival and transmission, thus contributing to significant economic losses for local producers [12].

### Role of contaminated water sources in transmission

The use of Jagüey reservoirs as water sources emerged as a notable risk factor. These open-air water reservoirs are susceptible to contamination with urine from infected animals, creating direct routes for transmission to cattle [51]. These findings highlight the importance of shared and stagnant water sources in the maintenance and spread of *Leptospira* within backyard systems.

### Public health relevance and one health perspective

There is a broad scientific consensus that leptospirosis is a neglected zoonotic disease, often underestimated, and transmitted to humans through contact with infected urine, as well as through soil and water contamination. Routine screening and early detection strategies can help mitigate its spread, especially among occupationally exposed populations such as farmers and herders [6], and reduce the risk of co-infections [52, 53].

### Addressing disease in vulnerable regions

The elevated incidence of leptospirosis in marginalized, tropical regions underscores the need for greater institutional attention. These areas are particularly vulnerable due to fluctuating environmental conditions, socioeconomic disparities, and insufficient veterinary infrastructure. As such, future research should assess the impact of public health policies on leptospirosis control using a One Health framework [6].

### Value of observed trends and future directions

Although not all associations identified in this study reached statistical significance, the observed epidemiological trends offer valuable guidance for the design of targeted preventive measures. Future studies should aim to validate these findings in broader, more diverse cattle populations across different geographic regions and management systems. This will be essential to improve risk stratification and develop effective, region-specific intervention strategies for leptospirosis control.

## CONCLUSION

This study provides the first comprehensive seroepidemiological assessment of *Leptospira* spp. in backyard cattle herds in the central region of Chiapas, Mexico. The overall seroprevalence was 27.72%, with *L. interrogans* serovar Canicola Portland Vere emerging as the most prevalent (22.89%), followed by Bratislava (5.51%). A striking 89.47% of PUs had at least one seropositive animal, emphasizing the widespread presence of *Leptospira* in these traditional cattle systems.

Significant associations were observed between seropositivity and several risk factors, including the use of artificial insemination, presence of dogs, type of water source (Jagüey and well water), animal age (notably 3 and 8 years), and breed type, particularly the American Swiss × Holstein cross. In addition, the spatial distribution analysis revealed higher seroprevalence in municipalities such as Cintalapa and Ocozocoautla, which are characterized by high rainfall, humidity, and cattle density, conditions that support *Leptospira* survival and transmission.

These findings highlight the importance of integrating targeted surveillance and control strategies in backyard PUs, including improved water source management, reproductive health monitoring, and tailored vaccination programs. Awareness campaigns and diagnostic outreach should be prioritized in high-risk zones to minimize economic losses and zoonotic spillover.

A major strength of this study is its region-specific focus on backyard cattle systems, a largely understudied sector in leptospirosis research, along with its use of spatial analysis, serovar-level identification, and multivariate modeling. The application of a One Health lens ensures a holistic understanding of disease dynamics at the livestock–environment–human interface.

While the study presents robust serological and risk factor data, limitations include the absence of longitudinal sampling and limited serological testing in companion animals (e.g., dogs), which may play a key role in transmission. In addition, a cross-sectional design limits inference of causal relationships.

Future studies should incorporate longitudinal monitoring to assess seasonal variations in seroprevalence, molecular typing to confirm infecting strains, and parallel surveys in dogs, wildlife, and humans to elucidate transmission pathways. Integrating climate and land-use data may further refine predictive models for leptospirosis outbreaks in tropical regions.

The seroprevalence of *Leptospira* in backyard cattle of Chiapas reflects a complex interplay of environmental, management, and biological risk factors. Given the zoonotic potential and economic burden of the disease, this study reinforces the urgent need for integrated control strategies aligned with One Health principles. A proactive approach to surveillance and biosecurity in backyard herds will be critical to safeguarding animal productivity and public health in vulnerable rural communities.

## AUTHORS’ CONTRIBUTIONS

LDRVN and GUBT: Conceptualized and designed the study. LDRVN, EHL, JLGH, and CACG: Collected samples and conducted laboratory analyses. LDRVN, JDCRO, EHL, JLGH, FACV, LMS, CACG, JAGM, RERE, and GUBT: Performed data analysis and interpretation, and drafted the manuscript. GUBT, FACV, LMS, and RERE: Conducted spatial analysis and mapping. All authors actively participated in revising the manuscript and approved the final version for publication.
